# World Trade Center Disaster and Asthma Type

**DOI:** 10.1289/ehp.12384

**Published:** 2009-02

**Authors:** Grace Ziem

**Affiliations:** Occupational and Environmental Health, Emmitsburg, Maryland, Website: chemicalinjury.net

As a practicing occupational health physician both in prevention and clinical care, I deeply appreciate the information provided by *EHP*. The article “Respiratory and Other Health Effects Reported in Children Exposed to the World Trade Center Disaster of 11 September 2001” (Thomas et al. 2008), published in the October 2008 issue of *EHP,* is consistent with current knowledge about adult consequences of the disaster. My concern is that the term “asthma” can be used to describe two clinical conditions that are not similar in either causation or treatment.

Allergic asthma, usually IgE mediated, is a separate entity clinically from irritant-induced “asthma.” The latter is often referred to as reactive airway disease because it is induced by exposure to irritants that may or may not be particulate, and irritant avoidance is an important component of treatment. Allergic asthma responds much better to bronchodilators than reactive airway disease. The latter has been described as having a nitric oxide/ peroxynitrite vicious cycle perpetuating biochemical mechanism. Irritant propellants used for treatment of allergic asthma often exacerbate reactive airway disease, and even relatively nonirritating bronchodilators such as albuterol, which help allergic asthma, do not help reactive airway disease.

I believe that it would be useful for Thomas et al. to clarify which condition they are describing: Given the exposure, irritant-asthma (also called reactive airway disease) appears to be the entity under study.
